# Development of In Vitro Corneal Models: Opportunity for Pharmacological Testing

**DOI:** 10.3390/mps3040074

**Published:** 2020-11-02

**Authors:** Valentina Citi, Eugenia Piragine, Simone Brogi, Sara Ottino, Vincenzo Calderone

**Affiliations:** 1 Department of Pharmacy, University of Pisa, Via Bonanno 6, 56126 Pisa, Italy; valentina.citi@unipi.it (V.C.); eugenia.piragine@farm.unipi.it (E.P.); vincenzo.calderone@unipi.it (V.C.); 2 Farmigea S.p.A., Via G.B. Oliva 6/8, 56121 Pisa, Italy; s.ottino@farmigea.it

**Keywords:** 3D in vitro models, eye research, in silico analysis, eye anatomy

## Abstract

The human eye is a specialized organ with a complex anatomy and physiology, because it is characterized by different cell types with specific physiological functions. Given the complexity of the eye, ocular tissues are finely organized and orchestrated. In the last few years, many in vitro models have been developed in order to meet the 3Rs principle (Replacement, Reduction and Refinement) for eye toxicity testing. This procedure is highly necessary to ensure that the risks associated with ophthalmic products meet appropriate safety criteria. In vitro preclinical testing is now a well-established practice of significant importance for evaluating the efficacy and safety of cosmetic, pharmaceutical, and nutraceutical products. Along with in vitro testing, also computational procedures, herein described, for evaluating the pharmacological profile of potential ocular drug candidates including their toxicity, are in rapid expansion. In this review, the ocular cell types and functionality are described, providing an overview about the scientific challenge for the development of three-dimensional (3D) in vitro models.

## 1. Introduction

The human eye is a deeply specialized organ with a singular anatomy and physiology, comprehending several structures with specific physiological functions. Due to the complexity of the eye, ocular tissues are finely organized and orchestrated. As a result, optimal visual function is maintained while the passage of solutes, fluids, and also drugs is highly controlled [1]. Briefly, the human eye is characterized by three main layers, which enclose many anatomical structures. The outermost layer is the fibrous tunic, composed of the cornea and sclera. The cornea and opaque sclera, its non-transparent extension, are inelastic structures that provide mechanical support to the eye globe, also protecting the eye from the external environment [2,3,4]. Moreover, the cornea is covered by the tear film, whose composition ensures hydration, provides nutrients, and further limits the entering of toxins or particles into the eye [3,5,6,7]. The middle layer (uvea or vascular tunic) includes the iris, pigmented epithelium, choroid, and ciliary body [8]. Finally, the innermost layer of the eye is represented by the retina, which is a neurosensory structure fundamental for the vision process [9,10,11]. According to its crucial role in regulating the vision process, many pathological conditions affecting the retina may progressively lead to an altered vision or blindness [12,13].

Based on these observations, along with the necessity to reduce tests on animals for evaluating the pharmacological profile of possible ocular drug candidates for given ophthalmic disorders (drug delivery/drug efficacy), including possible toxicity issues, the development of suitable and robust in vitro ocular models is a challenging task. These models allow to investigate the different aspects of the ocular pathophysiology of different diseases as well as the potential efficacy of possible therapeutic agents [14]. Furthermore, the use of these in vitro tools can be relevant for studying cell surface biomarkers for drug delivery. In the last years, along the ocular in vitro models, isolated primary cultures are expected to reproduce in vivo cellular functions and morphology in a more accurate way; however, these kinds of cells are difficult to cultivate since they arrest their growth quickly. Moreover, considering the human primary cells, it is very problematic to obtain numerous isolates for the restricted availability of human donor eyes. In order to overcome this issue, several attempts aimed at exploiting immortalized cell lines have been described to be used for pharmacological and biological investigations [15]. Unfortunately, the immortalized cell lines are characterized by altered gene expression patterns that often do not reflect the comportment of ocular cells in vivo, partially lacking the ability to mimic the complexity of the physiology of the human eye. However, the development of improved ocular cell-based models established also by reconstructing ocular tissues is fundamental for speeding up the discovery of safe ocular drugs with a relevant pharmacological profile. In this review, we report the most advance in vitro ocular models along with the computational approaches in the field of ophthalmic research. In fact, in the next sections, actual in vitro ocular models are discussed in detail, considering conventional two-dimensional (2D) models and advanced corneal three-dimensional (3D) models, with a particular focus on the application of the human cornea-like epithelium system and the potential models resembling human corneal diseases such as the zebrafish ocular surface. In addition, possible pharmacological application of 3D reconstructed human corneal tissues are reported as well as the most advanced in silico approaches in the field of ocular pharmacology and toxicology.

## 2. In Vitro Ocular Models

### 2.1. Opportunity and Application

**Figure 1:**
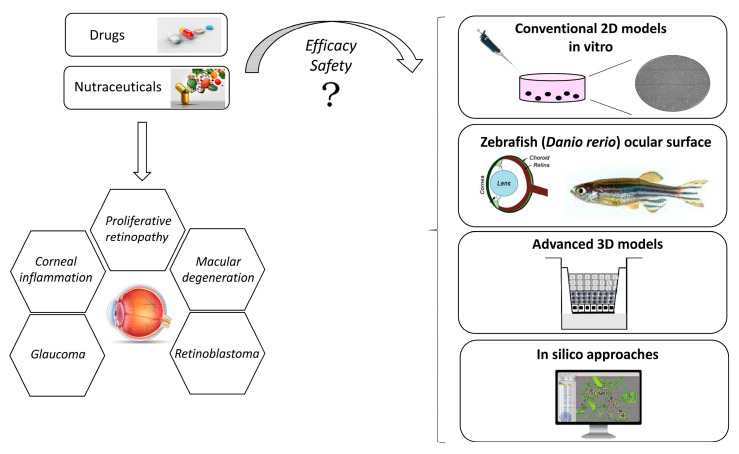
Relevant disease areas and the main model systems actually used for evaluating efficacy and/or safety of drugs and nutraceutical products.

Due to the complexity of the eye anatomy, a crucial issue in the development and realization of ophthalmic products and medical devices is to identify the specific mechanism of toxicity that could lead to severe adverse effects [6]. For this reason, recognizing and classifying the potential risk of commercial products is highly recommended to clearly know possible side effects. Eye toxicity testing is therefore necessary and mandatory to ensure that risks associated with the use of specific ophthalmic products follow appropriate safety criteria. In vitro preclinical testing is nowadays a well-established and important experimental approach for evaluating the efficacy and safety of cosmetic, pharmaceutical, and nutraceutical products (Figure 1) [16]. The realization and development of increasingly sophisticated experimental models, especially those based on reliable 3D cell cultures, can reduce the costs of experimental procedures, obtaining predictive information on the ocular tolerability and efficacy of a given product, severely limiting the in vivo experimentation on animals [17].

The development of novel in vitro approaches is firstly linked to the campaigns carried out in this decade by few associations, which strongly ask for a significant reduction in animal testing [18], leading to finding alternative methods and solutions to animal testing in the cosmetic, pharmaceutical, and nutraceutical fields. In particular, the principle of the 3Rs (Refine, Reduce, Replace) has been considered a stimulating opportunity to improve in vitro methods, even if nowadays it is not possible to completely abolish animal experimentation [19]. The scientific world gave the introduction of experimental in vitro models a strong impulse, implementing European Union (EU)-validated alternative methods in compliance with Good Laboratory Practice (GLP). In addition, the new Medical Devices Regulation (MDR 745/2017) is a further opportunity for companies operating in the preclinical sector [20] stimulating the exponential evolution of in vitro technologies. In particular, the potential of 3D systems (3D human tissues reconstructed in vitro) often proved to be more relevant and predictive than monolayer cell models. Many 3D models, at first, were quickly developed under the regulatory push, in order to replace animal models. They have been included in numerous OECD (Organisation for Economic Co-operation and Development) validation studies. In a short time, they became increasingly predictive with respect to the evaluation of a possible drug candidates, and were rapidly adopted in preclinical research. The advantages of using experimental in vitro 3D cell cultures models is due to their complex organization and structure, which is very similar to in vivo tissue [21], showing reproducible results in pharmacological and toxicological responses to reference substances.

The sections below aim at highlighting the substantial differences between conventional 2D models and advanced corneal 3D models, describing specific test guidelines already adopted for the evaluation of important toxicological and pharmacological responses.

### 2.2. Conventional 2D Models

The investigation of basic developmental or differentiation processes can be studied using primary or immortalized human cells deriving from the cornea, retina, and conjunctiva to understand and clarify pathophysiological conditions or to set up models in order to reproduce specific disease models and to perform toxicological and pharmacological studies [22]. Epithelial cells, keratocytes, fibroblasts, and trabecular meshwork cells are critical components required for the normal function of the ocular cell system. Atypical cell proliferation and regulation within the ocular cell system contributes to the development of disorders such as corneal inflammation, proliferative retinopathy, macular degeneration, glaucoma, and retinoblastoma. Cell culture models allow to evaluate the physiology of the different ocular cell types outside the living organism in reproduced conditions that mimic, as closely as possible, the environment of the tissue or organ from which they derive [23]. Among the possible applications, we can mention: (a) the investigation of the physiological processes of the cell life and of the response to exogenous treatments in a controlled environment [24]; (b) the evaluation of the effect of various molecules and drugs on specific cell types; and (c) the study aimed at generating reconstructed tissues (e.g., artificial corneal tissues). In the living organisms, cells are kept vital thanks to the supply of nutrients, supported by the vascular system which, through the capillary vessels, nourishes the tissue and abolishes those harmful molecules deriving from the cell metabolism. In vitro, the role of the vascular system is substituted by the culture medium, a highly nutritious liquid medium. It contains fundamental substances, such as glucose, amino acids, vitamins, and minerals, absolutely necessary for the physiological processes of cells, and animal serum, which supports cell growth and proliferation. Thanks to these culture conditions, ocular cell-culture models offer several advantages over animal experimental models, including a higher reproducibility, easier handling, and reduced costs, but still giving the possibility to study mechanistic processes of physiological or pathological altered pathways. Corneal cells can be directly exposed to test samples (chemicals or environmental matrix samples) at low and relatively defined concentrations [14]. In this regard, although distribution and excretion phenomena (which occur in in vivo exposure) do not occur, the bioavailable concentration of the test sample must be taken into account even in the in vitro models. The interaction of the sample with the cells allows a very rapid evaluation (even by hours) of the effect on cell activities and also allows to verify the reversibility of the response. Animal cell cultures can be used as a low-cost, rapid screening tool for toxicological and pharmacological evaluation of chemicals. Moreover, the problems deriving from interspecies variability are avoided if cells of human origin are used [25]. However, primary cells usually can only be used for limited passages before starting to lose their normal physiology and structural characteristics. Immortalized cell lines can be used for several passages, but they show the likelihood of developing chromosomal abnormalities, reduced expression of key markers, or abnormal growth [24]. However, there are also some limitations in the use of cell cultures. The in vivo–in vitro translation causes the loss of specific cell–cell interactions, histological characteristics of the tissue of origin and the components involved in homeostatic regulation (especially those of the nervous and endocrine systems). There are also metabolic alterations with a drop in some enzymatic levels (e.g., cytochrome P450) or changes in metabolic cycles, so that the energy metabolism of cells is largely based on glycolysis. Due to the strong selection in favor of the most actively proliferating cells, the culture also suffers a loss of differentiated properties.

### 2.3. Advanced Corneal 3D Models

**Figure 2:**
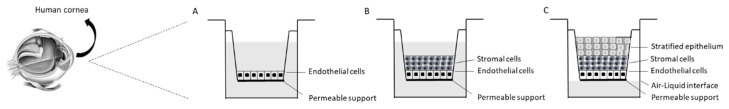
Schematic representation of a 3D in vitro corneal model: (**A**) Corneal endothelial cells grow on a permeable support up to confluence. (**B**) A 3D matrix containing stromal cells grows on top of the endothelial layer. (**C**) Epithelial cells are seeded on the stromal layer; then, exposure to air–liquid interface results in a stratified epithelium.

To date, regarding ocular studies, there are several in vitro methods that have been developed. Some of them suffer from diverse drawbacks, as in the case of organotypic and cell-based testing methods, that are scarcely compatible with real human eyes. Moreover, differences between species caused by the use of animals’ eyes may lead to an excessive and insufficient prediction of the eye irritation. The monolayer cell cultures employed in in vitro testing do not realistically reproduce the complicated 3D environment of real ocular tissues. Artificially rigid and flat surfaces of culture plates may alter cell metabolism and intrinsic functionality. To overcome these inaccuracies, 3D models equivalent to the human cornea have been developed based on normal human cells which are grown on an inert polycarbonate insert (Figure 2).

These tissues are validated and standardized, and each batch is derived from a single donor, giving a huge advantage in terms of accuracy and reproducibility. The human cornea is formed by the epithelium, stroma, and endothelium. Although ideal 3D models equivalent to human cornea should have all the three components of the cornea, only the human cornea-like epithelium (RhCE) has been presently developed, due to technical limitations. However, the corneal epithelium is the most important part to assess eye irritation, because it represents the outermost layer of the cornea, which protects the underlying tissue by excluding foreign material. There are a large variety of corneal models used to predict eye irritation including EpiOcular™, SkinEthic HCE, the Labcyte Cornea model, and MCTT HCE™. In particular, with regard to the reconstructed corneal tissue, the cells form a stratified and well-organized epithelium that is structurally, morphologically, and functionally similar to the human cornea presenting basal, wing, and mucosal cells [26]. These models are used to study drug delivery, as they represent a metabolically active tissue with the presence of tight junctions, characteristics of the human corneal epithelium. In addition, it has been shown that this type of tissue can be stimulated for the release of cytokines characteristic of an inflammatory state.

#### 2.3.1. Application of Human Cornea-Like Epithelium: Irritation Test Following OECD TG 492

The assessment of serious eye damage/eye irritation has typically been carried out by the use of laboratory animals. The development of novel 3D reconstructed human corneal tissues gave the opportunity to consider this substrate as a valid alternative for the setup of innovative experimental procedures in order to investigate important toxicological parameters. In particular, in the last few years, the corneal irritation assessment test guideline has been validated by using 3D human reconstructed corneal tissue following the OECD procedures [27]. This Test Guide, named OECD TG 492, describes an in vitro procedure that allows the identification of toxic and harmful chemicals (substances and mixtures) not requiring classification for eye irritation or serious eye damage by using human reconstructed corneal tissues [28]. These tissues closely reproduce the main feature of in vivo human cornea since they show physiological, morphological, histological, and biochemical properties of the human corneal epithelium. The RhCE tissues are produced from human immortalized corneal epithelial cells, primary human epidermal keratinocytes or primary culture human corneal epithelial cells, which are kept in controlled condition of temperature for several days to generate a highly differentiated, multi-stratified squamous epithelium morphologically similar to the human cornea [29]. However, it is currently accepted that no single in vitro test method will be able to fully replace the in vivo Draize eye test to predict serious eye damage/eye irritation responses for different chemical classes. Anyway, strategic combinations of several alternative test methods may be able to fully replace the Draize eye test. Moreover, the test method directly measures cytotoxicity resulting from exposure of the tested substance through the cornea and evaluates cell and tissue damage following chemicals exposure. Cell damage can occur due to several toxicological mechanisms, but cytotoxicity plays a fundamental role in determining clinically relevant eye damage response due to a given chemical entity [30]. Such a toxicity may lead to iritis, corneal opacity, conjunctival chemosis, and redness. The main cytotoxic mechanisms depend on the nature of the chemical exposure. In particular, cell membrane lysis can be caused by organic solvents and surfactants [31]; coagulation of macromolecules can occur if the eye is exposed to surfactants, organic solvents, alkalis, and acids [32]; saponification of lipids is mainly due to alkalis; alkylation or other covalent interactions with macromolecules are triggered by bleaches, peroxides, and alkylators. Furthermore, the serious eye damage/eye irritation effect of a toxicant is mainly associated with the extent of initial injury, which is related to the amount of cell death and with the magnitude of the following responses and eventual outcomes [33]. Thus, slight irritants generally only affect the superficial corneal epithelium, the mild and moderate irritants principally damage the epithelium and superficial stroma, while severe irritants are harmful for the epithelium, deep stroma, and the corneal endothelium. This test allows the use of different commercially available corneal-like tissues, in particular, the Labcyte CORNEA-MODEL24, EpiOcular™, and MCTT HCE™ RhCE tissue constructs that consist of at least 3 viable layers of cells and a nonkeratinized surface, showing a corneal-like structure similar to that found in vivo. The SkinEthic™ HCE RhCE tissue construct consists of at least 4 viable layers of cells including transitional wing cells, columnar basal cells, and superficial squamous cells similar to that of the normal human corneal epithelium. The measurement of viability of the RhCE tissues, after topical exposure to a chemical entity, is commonly used to identify its potential toxicity, carried out by enzymatic conversion of formazan salts (MTT assay) by the viable cells of the tissue into colored formazan salt, which is quantitatively measured after the extraction from tissues [34].

#### 2.3.2. Zebrafish Ocular Surface: A Model for Human Corneal Diseases?

The zebrafish (*Danio rerio*) is a common aquarium fish that originated in the Ganges region of India. The transparent embryos developing ex utero promoted a rapid increase in its popularity for the study of vertebrate development and genetics, making the visualization of developmental events reliable [35]. Detailed characterization of the embryonic development of the posterior segment of the eye, which includes the neural retina, the retinal pigment epithelium (RPE), and the anterior segment, highlighted the similarities in the architecture of the zebrafish eye to that of the human eye [36]. However, beside enormous similarity with the human eye, an interesting analysis about the structure of zebrafish eye structure and characteristics has been carried out by Puzzolo and colleagues [37]. The authors demonstrated that the corneal epithelium was formed by five layers of cells, but no Bowman’s layer has been reported. Moreover, the stroma is formed by lamellae of different thicknesses with few keratocytes [38]. The Descemet’s membrane was not detected. The immunohistochemical experimental procedures did not highlight corneal nerve fibers. The conjunctival epithelium was stratified and overlies the stroma formed by a subepithelial and a deep layer. This latter resulted to be connected to the scleral cartilage. The morphometric study demonstrated that the peripheral cornea epithelium was thicker if compared to the other parts of the ocular surface, with smaller superficial cells. The stroma was thinner in the conjunctiva than in the cornea, while corneal lamellae were thicker in the intermediate stroma. In conclusion, after a very interesting study, the authors concluded that the zebrafish ocular surface shows significant differences compared to the human, such as the absence of the Descemet’s membrane, Bowman’s layer and corneal nerve fibers, the presence of rodlet cells, and the reduced stromal thickness. Although the use of the zebrafish model is useful to predict eye toxicity after chemical exposure, such differences underline that the use of the zebrafish as a model for studying normal or pathological human corneas should be used with particular caution [37].

### 2.4. Pharmacological Application of 3D Reconstructed Human Corneal Tissues: The Dry Eye Model

Dry eye syndrome is caused by chronic dehydration of the conjunctiva and cornea, which induces irritation [7]. It is mainly due to a quantitative reduction or qualitative alteration of the tear film, which physiologically covers, lubricates, and protects corneal tissue [39]. Poor production or excessive evaporation of tears can be a complication of blepharitis, conjunctivitis (including allergic forms), and other inflammatory eye diseases [40]. Dry eye syndrome can also result from systemic diseases such as systemic lupus erythematosus and rheumatoid arthritis. Moreover, the disorder is typical in elderly patients (for the atrophy of the tear glands), in menopausal women (for the new hormonal balance), and in those who wear contact lenses [41]. Dry eye can be also related to iatrogenic causes for the use of several systemic drugs (antihypertensives, anxiolytics, sleeping pills, antihistamines). The most common symptoms due to dry eye syndrome are itching, burning, irritation, and annoyance to light (photophobia). In addition, a sensation of a foreign body pulling and scratching inside the eye, blurring of vision, difficulty opening the eyelid when waking up, eye pain, and hyperemia (red eyes) may also occur [42]. Tiredness or fatigue of the eyes may also appear and, in some patients, the appearance of mucus inside or around the eye is observed. All these disorders increase as a result of prolonged visual strain or under particular environmental conditions, such as exposure to wind or heat or staying in dusty, smoky, air-conditioned, or heated environments. In the most serious cases, the eye is exposed to increased friction due to eyelid movement and an increased risk of infections. In addition, it can degenerate to the appearance of lesions to the external structures of the eye: scarring, neovascularization, infections, and ulceration. Treatment for dry eye syndrome includes therapies that may vary depending on the cause and type of the disorder. Generally, medication is prescribed with eye drops or lubricating gels to help the eye to stay moist and clean [43]. When the patient’s eye allows it, it is also possible to prescribe protective contact lenses to protect the organ from rubbing with the eyelid. Developing novel medical devices for the treatment of dry eye syndrome is nowadays a challenging issue and 3D human corneal tissues have been recently used for the setup of in vitro dry eye model [44]. In particular, several research papers describe the realization of in vitro dry eye condition by exposing the tissues to specific conditions. Reconstructed corneal tissues are first treated with 0.6 M sorbitol in order to create a hyperosmolar environment mimicking the qualitative alteration of tear film. Furthermore, the tissues are exposed to 40 °C and 40% humidity for simulating the dryness [45]. After 24 h, tissues can be treated with the tested medical devices, and several biomarkers can be investigated. In particular, besides the tissue viability, also pro-inflammatory biomarkers (e.g., TNF, interleukins) and specific metalloproteinases which are responsible for corneal remodeling processes can be measured in order to characterize the efficacy of medical devices or drugs [46].

## 3. Computational Aspects for the Ocular Pharmacology and Toxicology

Along with in vitro models, for characterizing the pharmacological profile of possible drug candidates for treating ocular diseases, nowadays are growing different in silico approaches. These computational methods could be extremely useful for assessing the performance of given drug candidates saving money and time with respect to the drug discovery pipeline. In particular, computational pharmacology and toxicology represent a specified field of research comprehending in silico approaches for predicting, modelling, and explaining pharmacological effects and toxicological mechanisms at the molecular level. Several research studies have described the usefulness of in silico techniques for rapidly determining pivotal physico–chemical properties in order to optimize drug candidates (e.g., molecular weight, polarity, and lipophilicity). This computational evaluation is crucial for reducing off-target effects, and therefore the total number of animals required for the in vivo test. Computational pharmacology and toxicology take advantage from numerous scientific disciplines and usually include the application of in silico and statistical approaches for evaluating the bioactive profile of molecules for which a specific pharmacological or toxicological effect is not known, starting from a group of molecules for which the mentioned effect has been proven (training set) [47,48,49,50].

Accordingly, in silico strategies used for assessing the profile of compounds are mainly based on structure–activity relationship (SAR) and quantitative SAR (QSAR). In fact, most categories of computational methods in pharmacology and toxicology are based on the similarity principle: the hypothesis that compounds possessing a structural similarity could show comparable pharmacological or toxicological profiles. Numerous in silico techniques are commonly used for predicting both on- and off-target pharmacology of potential drug candidates [51,52]. Moreover, the use of computational approaches is decisive to limit animal testing also for the evaluation of potential ocular drugs and their possible toxicity. Currently, as mentioned above, the general evaluation of potential drugs is largely based on animal testing. In this context, the valuable advances in computational models are facilitating to amend this standard. First of all, the regulatory agencies are encouraging the usage of in silico toxicology models for accomplishing the growing public request in order to improve animal welfare. This latter has convinced governmental organizations to boost the reduction of animals used in in vivo tests, encouraging alternative procedures for evaluating promising potential drug candidates. This exigence is well enclosed in the 3Rs principle [53]. Accordingly, the computational tools employed to characterize a given set of compounds are almost without cost, and they are applicable for virtual molecules before their synthesis, limiting the use of animal in preclinical development, testing only the most promising computational hits. Classical QSAR analysis for determining potential pharmacological profiles has been amended for predicting general toxicity and ocular toxicity as well as the side effects of drugs, developing quantitative structure–toxicity relationship (QSTR) models [54]. This approach is widely used for generating models in order to computationally assess the potential toxicity of chemical entities. In QSAR approaches, the quality of the developed models is dependent on the chemical/molecular descriptors and the modelling strategies that are used. For example, early efforts about the QSAR modelling for predicting ocular toxicity were founded on the simple linear regression technique and empirical descriptors such as the physico–chemical properties [55,56]. These kinds of models are surely easy to explain and implement due to their simplicity, but their efficacy is restricted to molecules that are extremely similar to the molecules included in the training set. Later, more complex modelling strategies and descriptors have also been applied in this field of research. For example, it is possible to use membrane-simulated models for studying ocular toxicity, identifying a group of descriptors that appropriately correlate to the cornea permeability. The individuation of appropriate descriptors, as in the mentioned case, were also used for developing eye irritation models [57].

Furthermore, because only one type of descriptor and one modeling approach were used in most of the existent computational approaches, as the works centered on the Draize test data, the developed models for predicting ocular toxicity suffer from a difficult-to-predict toxicity for different structural unrelated chemical entities. This drawback is partially overcome by using an improved QSAR method such as the combinatorial QSAR (combi-QSAR) approach [58]. This technique relies on the use of numerous diverse combinations of many chemical descriptors and modelling strategies. By this approach, it is highlighted that the improvement of the number of descriptors is crucial for developing effective predictive models. Furthermore, combi-QSAR models can culminate in a consensus model (i.e., averaging of the results of all individuals) in order to improve predictivity and coverage [59]. The main drawback of these models is surely the lack of sensitivity and/or specificity combined with an inability to predict the exposure to a given drug that would elicit the adverse effects, making these models needing some improvements for their use as part of the ocular drug development trajectory. In general, these issues could be overcome by Machine Learning (ML)/Deep Learning (DL) approaches [60], but actually regarding the ophthalmology, DL has displayed clinically satisfactory diagnostic performance, but only for detecting various retinal pathologies. In fact, DL in ocular imaging may be employed in combination with telemedicine as a potential solution for screening, diagnosing, and monitoring main eye disorders (e.g., age-related macular degeneration, glaucoma, diabetic retinopathy, choroidal neovascularization, and other macular diseases) [61,62]. Accordingly, only few examples of ML application to ocular pharmacology and toxicology are available and often referred to one ocular toxicity condition [7,63]. On the other hand, attempts are starting to appear in literature to model ocular traumas. In this context, eye injuries referred to the interaction of a blast wave with ocular tissues are defined as primary ocular blast injury (POBI). Interestingly, a method for investigating POBI has been described. For this purpose, a finite element model of the human eye employing simple constitutive models was developed, taking into account material parameters adjusted using a multi-objective optimization accomplished on existing eye impact test information. Using this strategy is possible to model the behavior of the human eye and the dynamics of mechanisms occurring under POBI loading conditions, predicting the human eye reaction under diverse kinds of shocks. This approach is useful for the development of in silico models in order to understand processes causing ocular tissue injuries [64].

In view of that, based on the previous discussion, it is possible to predict, in the next years, a rapid growth of computational approaches in this field. The future aim will be turned at reaching a significant improvement and robustness of in silico models regarding ocular pharmacology and toxicology.

## 4. Conclusions and Future Perspective

Thanks to the intense efforts that have recently been implemented by biomedical research in in vitro alternative methods, 3D corneal tissue models are becoming a real prospective of alternative experimental models, in particular to Draize test, which has been extremely criticized for ethical motivations. Based on the previous discussion, it is possible to predict, in the next years, a rapid growth of both the 3D tissue model and computational approaches aimed at reaching a significant improvement and robustness of in vitro models regarding ocular pharmacology and toxicology. Furthermore, although not yet approved by OECD testing guidelines, in the last few years, more innovative organoid (or organ-on-a-chip) in vitro models have been created. The development of this technology based on microfluidics closes the gap between in vitro and in vivo models by offering new approaches for pharmacological research. In fact, organs-on-chip can combine both preclinical models previously discussed, cultivating human cells in tissue-specific 3D contexts. The advantage is that 3D cell culture models promote higher levels of cell differentiation and tissue organization than the usual 2D models.
